# Spatial stochastic modelling of the Hes1 gene regulatory network: intrinsic noise can explain heterogeneity in embryonic stem cell differentiation

**DOI:** 10.1098/rsif.2012.0988

**Published:** 2013-03-06

**Authors:** Marc Sturrock, Andreas Hellander, Anastasios Matzavinos, Mark A. J. Chaplain

**Affiliations:** 1 Department of Mathematics, University of Dundee, Dundee DD1 4HN, UK; 2 Department of Computer Science, University of California, Santa Barbara, CA 93106, USA; 3 Division of Mathematics, Iowa State University, Ames, IA 50011, USA

**Keywords:** Hes1, spatial stochastic modelling, negative feedback loop, simulation

## Abstract

Individual mouse embryonic stem cells have been found to exhibit highly variable differentiation responses under the same environmental conditions. The noisy cyclic expression of Hes1 and its downstream genes are known to be responsible for this, but the mechanism underlying this variability in expression is not well understood. In this paper, we show that the observed experimental data and diverse differentiation responses can be explained by a spatial stochastic model of the Hes1 gene regulatory network. We also propose experiments to control the precise differentiation response using drug treatment.

## 1. Introduction

Many gene regulatory networks (GRNs) exhibit oscillatory dynamics in space and time in response to a range of external stimuli [1–4]. A negative feedback loop often lies at the core of such networks, controlling the levels of mRNA and proteins. These proteins are usually transcription factors, which initiate or regulate transcription in eukaryotic cells, and in order for them to function they must bind to specific DNA sequences in the nucleus. One striking example of a regulatory network containing a negative feedback loop is the Hes1 GRN. The Hes1 GRN plays a central role in the timing of somitogenesis [1] and can become deregulated in human cancer [5].

There are numerous sources of stochasticity and heterogeneity in biological systems, and these can have important consequences for understanding the overall system behaviour. Intrinsic noise is commonly found in many intracellular signalling pathways [6–8]. This noise can arise as a result of low abundance of molecular species, randomness in certain key processes (e.g. binding and unbinding of transcription factors to promoter sites), stochasticity in production processes (transcription and translation) and degradation events [9].

In addition to being inherently stochastic, intracellular signal transduction is inherently spatial. The eukaryotic cell hosts a variety of spatial compartments (e.g. cytoplasm, endoplasmic reticulum, Golgi apparatus, nucleus, mitochondrion, etc.). Each compartment permits different metabolic activity and is often separated from the rest of the cell by a thin lipid membrane. Signalling molecules reach the appropriate spatial compartments through molecular movement, such as diffusion and active transport. The key process of transcription occurs at highly localized sites, for example, genes, in the nucleus. Within the cytoplasm, another key process, such as translation occurs in the ribosomes. Clearly, mathematical models of GRNs will be more realistic the more they seek to account for stochastic and spatial features of these networks.

Very few spatial stochastic models exist in the literature but this is beginning to change. Some of the first spatial stochastic models were of the Min System in an *Escherichia coli* cell [10,11]. Howard & Rutenberg [10] used a stochastic analogue of a one-dimensional system of reaction–diffusion equations and found that for some parameter values the protein concentrations were low enough that fluctuations were essential for the generation of patterns. In the model of Fange & Elf [11], trajectories were generated using the next subvolume method (NSM), and numerical simulations were able to reproduce all documented Min phenotypes, where deterministic or non-spatial models could not. A spatial stochastic model of the MAPK pathway was developed in the study of Takahashi *et al*. [12]. This model was implemented numerically using a Green's function reaction dynamics scheme, which allows for individual particle-level simulation of molecular species. Using this technique, MAPK responses that could not be observed using a mean-field approach were produced. Another recent spatial stochastic model was developed to study in detail a generic transcription factor binding and unbinding to DNA [13]. Here, the spatial stochastic model was able to support the use of well-stirred, zero-dimensional models for describing noise in gene expression. It is clear from these few examples that spatial stochastic modelling can provide insight into intracellular signalling pathways that other approaches cannot. For a comprehensive review of spatial stochastic modelling of intracellular processes, see the study of Burrage *et al*. [14].

The development of mathematical models which reflect both spatio-temporal and stochastic aspects of GRNs can be regarded as an important computational tool in making predictions about the behaviours of GRNs and in the optimizing of targeted drug treatment. In this paper, we propose a novel spatial stochastic model of the Hes1 GRN. We focus our study on Hes1 oscillations observed in embryonic stem (ES) cells, as the quality and abundance of Hes1 expression data for this cell line far exceeds all others.

## 2. The Hes1 gene regulatory network

**Figure 1.**
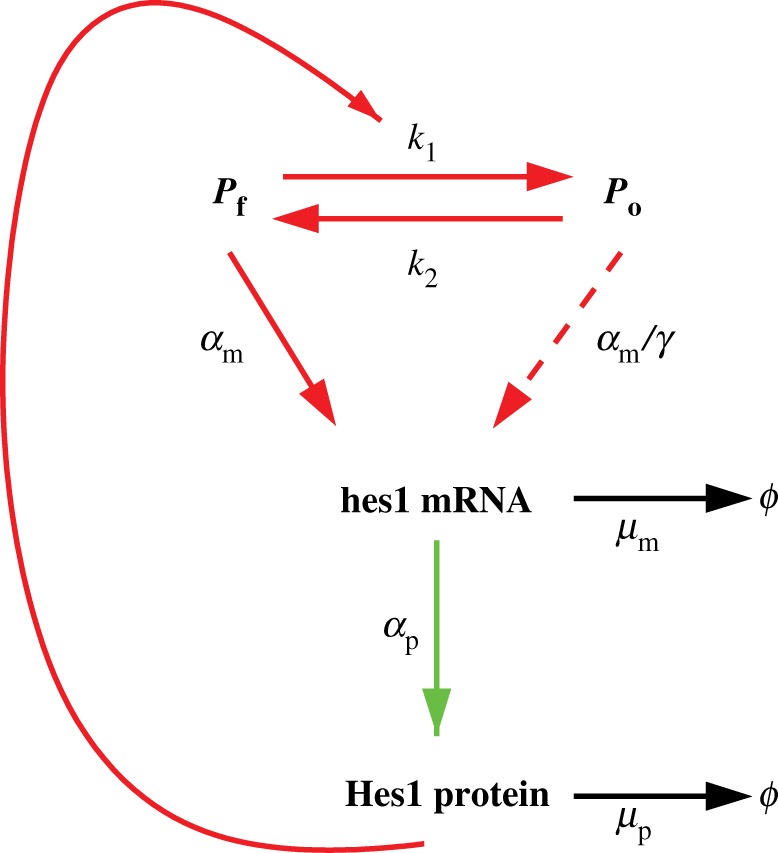
The negative feedback loop in the Hes1 GRN. When the promoter site is free, hes1 mRNA is transcribed at its maximal rate. hes1 mRNA then produces Hes1 protein via the process of translation. Hes1 protein occupies the promoter and represses the transcription of its own mRNA. The occupied promoter site is still able to produce hes1 mRNA, but at a significantly reduced rate [15]. Reaction arrows displayed in red only occur at the promoter site, while those in green occur only in the cytoplasm and those in black occur everywhere within the cell.

Hes1 is a member of the family of basic helix–loop–helix (bHLH) transcription factors. Hes1 is known to play a role in somitogenesis, the developmental process responsible for segmentation of the vertebrate embryo. During somitogenesis, a ‘segmentation clock’ controls the timing of the assignment of mesodermal cells to discrete blocks. The segmentation clock depends on the oscillatory expression of a complex network of signalling pathways, including the Hes1 GRN which contains a negative feedback loop (figure 1). This feedback loop is formed through interactions of the Hes1 protein with its own gene—Hes1 protein binds to N box sequences on the hes1 promoter and represses the transcription of hes1 mRNA.

Experiments have been conducted to measure expression levels of hes1 mRNA and Hes1 protein in many different cultured mouse cell lines [1]. In response to a single serum treatment, it was found that levels of hes1 mRNA and Hes1 protein exhibited oscillations with a regular period of approximately 2–3 h. This coincides with the period observed for the mouse segmentation clock. It has been found that Hes1 oscillations are stable (both the period and amplitude are relatively constant) in presomitic mesoderm cells but unstable (the period and amplitude are variable) in individual dissociated presomitic mesoderm cells, suggesting that cell–cell communication is essential for stabilization of such cellular oscillators [16]. Hes1 oscillations have also been observed in neural progenitor cells, again with a period of about 2–3 h [17]. It was found that these oscillations were responsible for the maintenance of neural progenitors and that sustained overexpression of Hes1 inhibits proliferation and differentiation of these cells. More recently, Hes1 expression was monitored in ES cells [18], where it was discovered that Hes1 levels still oscillated in space and time, but with a period of 3–5 h, longer than that of other cell lines. This lengthened period is thought to be a result of the increased stability of hes1 mRNA in ES cells. It has also been discovered that Hes1 oscillations contributed to heterogeneous differentiation responses of ES cells. Using fluorescence-activated cell sorting, ES cells with high and low expression levels of Hes1 were isolated and then immediately transferred to a neural differentiation medium. It was found that cells expressing low and high levels of Hes1 differentiated into neural and mesodermal cells, respectively [19,20].

Previous mathematical models of the Hes1 negative feedback loop have taken a variety of forms. The first model adopted an ordinary differential equation approach [1], while later models used delay differential equation (DDE) models [21,22]. The effect of low particle numbers in the DDE system was considered in [23], where the stochastic simulation algorithm (SSA) [24], extended to allow for delays, was used. Further modelling of the Hes1 oscillator found that there is little evidence for synergistic binding in the regulatory region of the Hes1 gene [25]. The role of Gro/TLE1 has also been considered [26] and other models have examined the role of the Hes1 pathway in somitogenesis [27]. Spatio-temporal models of the Hes1 negative feedback loop were presented in the study of Sturrock *et al*. [28], using a partial differential equation (PDE) model while extensions of this were considered in the study of Sturrock *et al*. [29].

### 2.1. A spatial stochastic model of the Hes1 gene regulatory network

The basic assumptions concerning the molecular reactions in the Hes1 feedback loop follow previous modelling efforts [21]. Figure 1 shows a schematic description of the network. Our model explicitly considers the spatial distributions of the species so reactions are now localized to separate compartments of the cell, as indicated by the colours of the arrows.

We assume that the promoter site exists in two states—a free state or one occupied by Hes1 protein, represented by *P*_f_ and *P*_o_, respectively. All reactions are modelled by elementary mass action kinetics. This is in contrast to all previous modelling efforts where a Hill function approximation was used for Hes1 binding to the promoter. Since our model is explicitly spatial, such an approach is neither appropriate nor necessary.

### 2.2. The reaction–diffusion master equation

To account for intrinsic stochasticity, we model the reaction–diffusion kinetics as a continuous time discrete-space Markov process. The state of the system is the discrete number of molecules of each of the species as a function of time. The likelihood of a transition is described by its reaction propensity, which defines the probability of transition from the state *x* to *x* + *N_r_* per unit time2.1
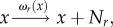
where 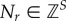 is the transition step and is defined as the *r*th column in the stoichiometric matrix *N* and *ω_r_*(*x*) is the reaction propensity function. When the system can be considered well mixed, the SSA [24] or variants of it are typically used to generate statistically exact realizations of the process.

**Figure 2.**
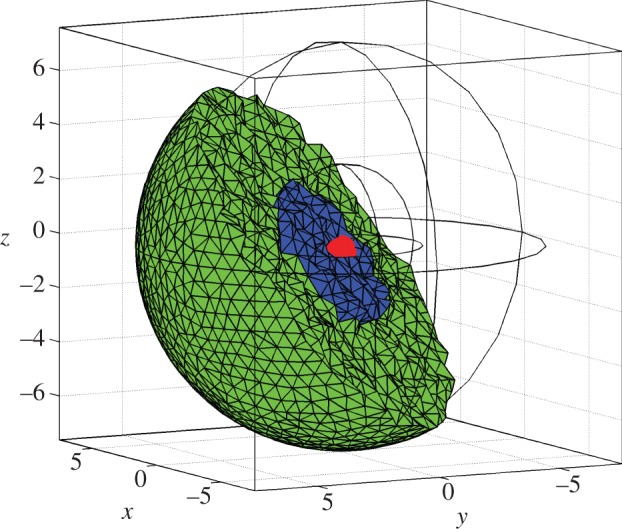
The three-dimensional meshed domain used in numerical simulations of the Hes1 model. The domain is discretized such that 10 946 voxels make up the domain. Here, the units of axes are in micrometres. The cell is represented by a sphere, centre (0,0), with a radius of 7.5 μm. The nucleus is shown as a blue sphere, centre (0,0), with a radius of 3 μm. The cytoplasm (shown in green) is the part of the cell that is outside the nucleus. The gene subdomain is chosen to be the voxel closest to the centre of the cell (0,0), a distance *r* from the nuclear membrane.

To introduce molecular motion owing to diffusion, the spatial domain is subdivided into non-overlapping voxels in a mesh (cf. figure 2). Diffusion is modelled as first-order events where a species *S_l_* in voxel *ψ_i_* moves to an adjacent voxel *ψ_j_*, i.e.2.2
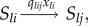
where *x_li_* is the number of molecules of species *l* in voxel *i* and *q_lij_* is a diffusion rate constant that depends on *D_l_*, the diffusion coefficient of species *l*, and on the size and shapes of voxels *ψ_i_* and *ψ_j_*. The equation that governs the time evolution of the probability density of the system is called the reaction–diffusion master equation (RDME). A more detailed description of the modelling framework can be found in the electronic supplementary material. We assume that both hes1 mRNA and Hes1 protein can diffuse as described above, with diffusion coefficient *D* = 6.00 × 10*^−^*^13^ m^2^ min*^−^*^1^ [30]. We do not allow promoter species to diffuse, rather we assume the promoter species remain in the gene subdomain.

For fine discretizations, the classical SSA becomes inefficient. NSM [31] is an algorithm adapted for simulations of the RDME, and it inherits good scaling properties from the next reaction method (NRM) [32]. For all following simulations, we have used NSM as implemented in the unstructured mesh reaction–diffusion master equation (URDME) software framework [33]. URDME uses unstructured tetrahedral and triangular meshes such as shown in figure 2, thus enabling simulations to be performed on complex geometries. The diffusion rate constants *q_lij_* are automatically computed for the unstructured mesh as described in more detail in the earlier studies [33,34].

### 2.3. Domain, initial and boundary conditions

The computational domain is shown in figure 2. The cell is represented by two concentric spheres with centre (0,0) and radius 7.5 and 3 μm, respectively. The inner sphere models the nucleus. These values are chosen to be consistent with experimental measurements of ES cells [35]. The promoter site, or gene subdomain, is taken to be a single voxel at a radial distance *r* from the nuclear membrane. Unless otherwise stated, we choose the promoter site to be at *r* = 3 μm, i.e. the voxel closest to the centre of the cell (0,0). We arbitrarily choose initial conditions such that 60 Hes1 proteins are uniformly distributed in the cytoplasmic subdomain, 10 mRNA molecules in the nuclear subdomain and a single free promoter is found in the gene subdomain (our model does not appear to be sensitive to initial conditions—see §6 of the electronic supplementary material). Zero-flux boundary conditions are applied at the cell membrane and continuity of flux boundary conditions are applied at the nuclear membrane as a means of modelling the transport in and out of the nucleus.

A summary of the reactions, their subcellular localization, and the initial parameters used in the simulations are found in table 1.

**Table 1.** Description of reactions in the Hes1 model, their localization and initial parameter values used.

reaction	description	localization	parameter values
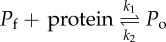	binding/unbinding of Hes1 protein to promoter	promoter site	*k*_1_ = 1.00 × 10^9^ M^−1^ min^−1^, *k*_2_ = 0.1 min^−1^
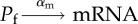	basal transcription of hes1 mRNA	promoter site	*α*_m_ = 3.00 min*^−^*^1^
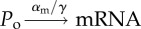	repressed transcription of hes1 mRNA	promoter site	*α*_m_ = 3.00 min*^−^*^1^, *γ* = 30.00
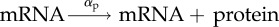	translation of Hes1 protein	cytoplasm	*α*_p_ = 1.00 min*^−^*^1^
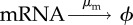	degradation of hes1 mRNA	entire cell	*μ*_m_ = 0.015 min*^−^*^1^
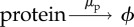	degradation of Hes1 protein	entire cell	*μ*_p_ = 0.043 min*^−^*^1^
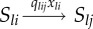	molecular diffusion	entire cell	*D* = 6.00 × 10*^−^*^13^ m^2^ min*^−^*^1^
	radial distance of gene from nuclear membrane	nucleus	*r* = 3 µm

## 3. Results

### 3.1. The model reproduces quantitative and qualitative behaviour of wild-type embryonic stem cells

**Figure 3.**
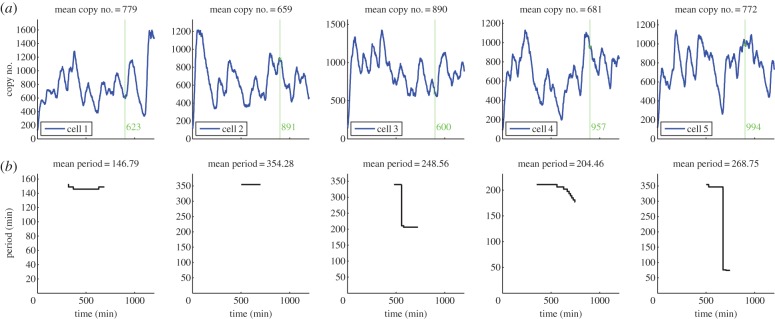
In row (*a*), plots of the total numbers of Hes1 protein (found by summing the number of proteins over the entire cell domain) are presented against time for five different trajectories of the Hes1 model. The mean copy numbers are displayed in the titles of row (*a*). The green vertical line represents the transference of cells to a neural differentiation medium. The number highlighted in green is the copy number of Hes1 at this time. Row (*b*) shows the corresponding time varying period as approximated by a Morlet continuous time wavelet transform with Gaussian edge elimination. The mean periods are displayed in the titles of row (*b*). Baseline parameter values are used (table 1).

We performed simulations of the Hes1 GRN model using the parameter values in table 1 and in order to be consistent with biological experiments, we ran our simulations for 1200 min [18]. Five representative trajectories are displayed in figure 3*a*, along with corresponding periods figure 3*b*. These time-dependent periods are estimated using a Morlet continuous time wavelet transform as implemented in WAVOS (for the most appropriate technique for these data, see [36] for details) and we use Gaussian edge elimination to minimize artefacts in the approximation of the period.

**Figure 4.**
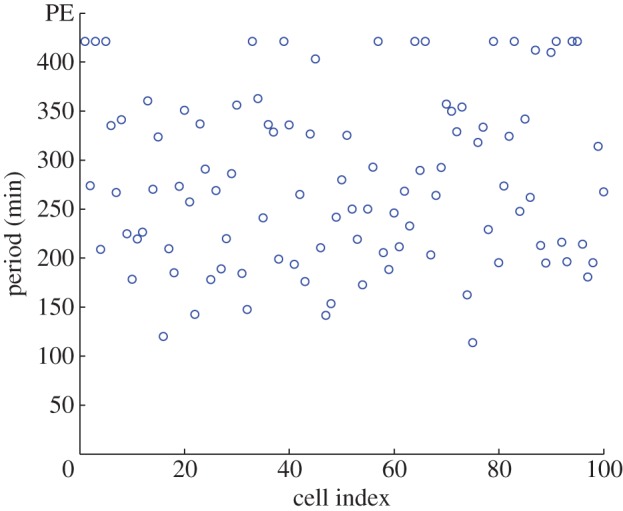
Plot showing the period of 100 different trajectories. The periods were calculated using a Morlet continuous wavelet transform with Gaussian edge elimination. Baseline parameter values are used (table 1).

The evolution of the total number of proteins is in close agreement with recent experimental studies, in terms of qualitative behaviour and quantitative values for the period. Although there have been many experiments performed to analyse the oscillatory nature of the Hes1 protein, it is not clear what units are used to measure protein expression levels, hence it is difficult to compare the numbers of Hes1 protein predicted from our model with real experimental values. However, we have received estimates of the copy number of hes1 mRNA in ES cells from experimentalists (see electronic supplementary material, table S3), which fall in the range 0–465, and our mRNA values also fall in this range (see electronic supplementary material, figure S1). Note that although there are large amplitude oscillations or variations in the protein copy number levels, the hes1 mRNA copy numbers are relatively stable. This phenomenon of small variations in mRNA copy number leading to large variations in protein copy number is consistent with other studies, see [37] for example. It is reasonable to assume that protein levels will be higher than mRNA levels, see [38,39], hence the values predicted by our model (figure 3) may be consistent with experimental values. Unlike the copy number of Hes1 protein, values for its period can be found in the literature. Experimentalists estimated that the period for Hes1 protein in ES cells lies in the range of 180–300 min. The periods from 100 different trajectories of our model are displayed in figure 4, and many of these lie in the same range reported by biologists (compare figure 4 with electronic supplementary material, figure S12). Since our model accounts for intrinsic noise, it is able to reproduce the highly variable period and amplitude found in the expression of Hes1 protein in ES cells. This is a feature that recent PDE models are not able to reproduce [28,29].

**Figure 5.**
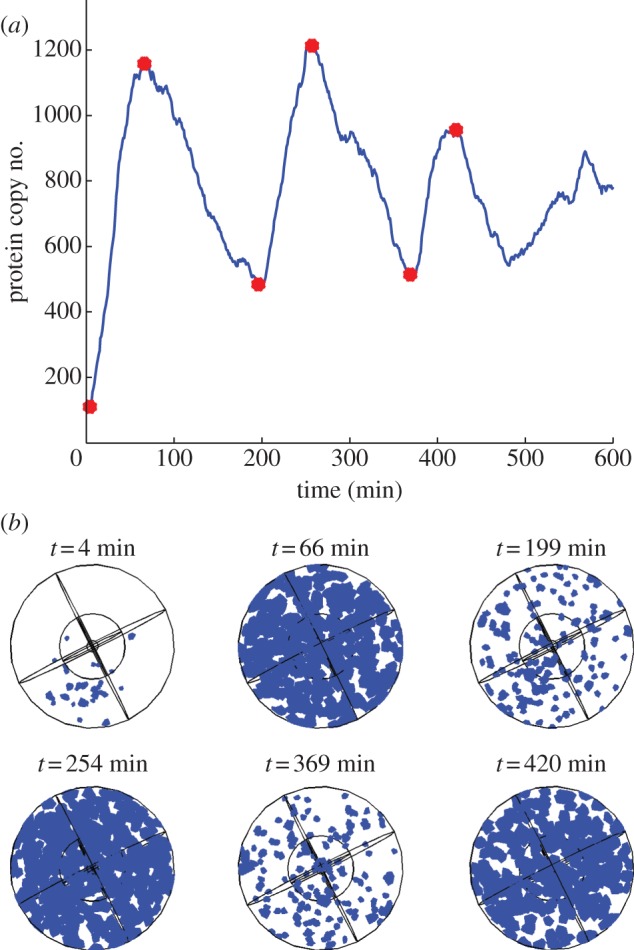
(*a*) Plot showing the total Hes1 protein copy number over a period of 600 min from a single trajectory of the Hes1 model (see table 1 of the main paper for parameter values), and (*b*) plots showing the corresponding spatial distributions of Hes1 protein. The times for these spatial snapshots were chosen to correspond to the peaks and troughs of oscillations in Hes1 protein copy number shown in (*a*) above. These times are highlighted by the red circles in (*a*). In (*b*) blue voxels indicate regions of the cell which contain Hes1 protein.

Furthermore, we include a plot of spatial snapshots of the spatio-temporal evolution of Hes1 protein in figure 5. Such spatial plots can be compared with experimental movie clips of bioluminescence imaging of Hes1 protein in ES cells (see supplemental movie file of [18], for example). We also include additional plots of the other model species in the electronic supplementary material. In addition to the total copy number of hes1 mRNA and Hes1 protein, the switch-like behaviour of the promoter states is clearly visible in the electronic supplementary material, figure S1.

### 3.2. Intrinsic noise can explain variability in embryonic stem cell differentiation

Our model produces some trajectories that either have a period that is unrealistically long (more than 400 min) or simply fail to oscillate with non-negligible amplitude. We shall label these trajectories as cells exhibiting ‘persistent expression’ (PE) of Hes1. For example, in figure 4, we can observe 15 trajectories falling into this category. In ES cells, as stated earlier, persistent high levels of Hes1 was indicative of cells that would differentiate into mesodermal cells. Hence, our model can yield predictions concerning the differentiation response of ES cells. In particular, given a batch of ES cells, it is possible to predict how many would differentiate into neural and mesodermal cells at a specific time. We have illustrated this idea in figure 3*a*. The green vertical line indicates the time at which cells are transferred to a neural differentiation medium (900 min) with the copy number of Hes1 at this time given beside the line. Cells with high expression of Hes1 protein at this time would differentiate into mesodermal cells while those displaying low expression levels would differentiate into neurons. If we define high and low expression as the copy number being greater than or less than the mean, respectively, then we suggest that of the trajectories displayed in figure 3, cells 2, 4 and 5 would differentiate into mesodermal cells and cells 1 and 3 would differentiate into neurons. Hence, by accounting for intrinsic noise, our simple model is able to reproduce the variability encountered experimentally in ES cell differentiation.

### 3.3. Hes1 exhibits highly variable expression levels under a wide range of conditions

Here, we explore the parameter space of our model in a bid to find the main sources of stochasticity and variability exhibited in its trajectories. We achieve this mainly through parameter sweeps. A parameter sweep is performed by holding all parameter values at their baseline values (table 1), then varying a single parameter over some finite range and recording 100 trajectories for each new parameter set produced. For each trajectory recorded, we compute its mean period (as in figure 4) and visualize the output in a histogram. We perform parameter sweeps for all parameters in the model and plot the histograms produced in the electronic supplementary material. We discuss here the two parameters for which we do not have experimental measurements, namely, *k*_1_ and *k*_2_ as well as two spatial parameters, *D* and *r*. Note that by only varying one parameter at a time, we are neglecting most of the parameter space. A future study will investigate the full parameter space of our model using data-clustering techniques.

In general, we found from the parameter sweeps that the model produces broad distributions of periods whenever oscillatory dynamics are found. Provided the sweep does not yield trajectories entirely exhibiting persistent expression of Hes1 then we find great variety in the mean periods computed.

#### 3.3.1. Hes1 must bind to the promoter sufficiently fast for oscillations to be observed

The rate at which Hes1 protein binds to the promoter region of the hes1 gene is an important parameter in our model. It is responsible for the negative feedback Hes1 protein exhibits on its own mRNA production. We vary *k*_1_ over the range (1.00 × 10^7^−1.00 × 10^10^)M*^−^*^1^ min*^−^*^1^, which is in line with experimental measurements of protein–DNA-binding rates [40]. The histogram displaying the mean periods from the parameter sweep of *k*_1_ is displayed in electronic supplementary material, figure S7. The results are consistent with intuition—if *k*_1_ is too small, Hes1 protein is unlikely to bind to the promoter site and so the majority of trajectories display PE. Experimentalists have compared the expression levels of wild-type Hes1 and a functionally defective Hes1 mutant, which is unable to bind to the N or E box DNA sequence, in haematopoietic progenitor cells. The authors reported no repression of Hes1 when the mutant levels were monitored, in contrast to the wild-type case [41]. This is comparable to low values of *k*_1_ in our model, which produces trajectories which mainly exhibit persistent expression (i.e. no repression of Hes1 levels). Hence, using our model, we can investigate both mutant and wild-type Hes1 genes. If we set *k*_1_ = 0, then all trajectories are found to display PE, with high values of protein. As *k*_1_ is increased, we obtain a broad range of periods, which appear to be quite robust to change provided *k*_1_ is above approximately 2.50 × 10^8^ M*^−^*^1^ min*^−^*^1^.

The parameter value for which we have the least information in our model is *k*_2_, the rate at which protein unbinds from the promoter site, making the promoter free again. We vary *k*_2_ in the interval 0.1−1 min*^−^*^1^ and the histogram containing this parameter sweep is displayed in electronic supplementary material, figure S8. For lower values of *k*_2_ (0.01–0.34 min^−1^), we can observe a broad range of periods, but as *k*_2_ is increased, we find more and more trajectories displaying PE of Hes1. This can be interpreted biologically as the promoter site becoming free too quickly, which would prevent the negative feedback from taking effect. As in the case of parameter *k*_1_, if we set *k*_2_ = 0, we find no oscillations in the trajectories of our model. However, in contrast to *k*_1_, we find low protein levels.

#### 3.3.2. Oscillatory dynamics are only found for sufficiently large diffusion coefficients

It was reported in [28,29] that PDE models of Hes1 oscillations exhibited oscillatory dynamics for a finite range of values of the diffusion coefficient, i.e. if the diffusion coefficient was too large or too small then oscillations ceased. We investigate a range of values for the diffusion coefficient in our model, in order to see whether the same properties are retained in our stochastic model (see electronic supplementary material, figure S9 for the corresponding parameter sweep). Interestingly, in the context of observing oscillatory dynamics, it appears that D is bounded below, but not above. No matter how large the diffusion coefficient is made, the model still yields oscillations. This is likely to be a result of the stochastic nature of our model. Even if the diffusion coefficient is very large, it is still not a certainty that the protein will find the gene site almost instantly, which is the case in the corresponding continuum model. However, if the diffusion coefficient is too small, then mRNA and protein will stay in the subdomain where they originated, which is reflective of the continuum case. Overall, our spatial stochastic model is more robust to changes in the diffusion coefficient than a continuum model of the same GRN. In particular, oscillatory dynamics are observed for any diffusion coefficient greater than or equal to *D* = 5.00 × 10*^−^*^14^ m^2^ min*^−^*^1^.

#### 3.3.3. Oscillatory behaviour is robust to changes in the position of the promoter site if the diffusion coefficient is large enough

It is known that some genes are located closer to the nuclear membrane than others, which increases their sensitivity to transcription factors [42]. Evidence of precisely where the Hes1 gene is located within the nucleus is lacking, and in any case this is likely to change from cell to cell. Hence, given the symmetry of our domain, we investigate the influence of varying the distance *r* of the promoter site from the nuclear membrane for three different diffusion coefficients (see electronic supplementary material, figure S10 for the parameter sweeps). For a low value of the diffusion coefficient (*D* = 1.00 × 10*^−^*^14^ m^2^ min*^−^*^1^), we find that the location of the promoter site strongly influences the oscillatory behaviour observed. Persistent expression of Hes1 is observed when the promoter site is placed further away from the nuclear membrane, and as the promoter site is moved closer to the nuclear membrane, we find a broader distribution of periods. A slight dependence on promoter site location is observed for the default value of the diffusion coefficient, *D* = 6.00 × 10*^−^*^13^ m^2^ min*^−^*^1^. Here, if the promoter site is too close to the nuclear membrane, more trajectories exhibiting PE are found. Finally, for larger diffusion coefficients, specifically *D* = 1.00 × 10*^−^*^11^ m^2^ min*^−^*^1^, we find a broad range of oscillatory dynamics which are robust to promoter site location.

### 3.4. Controlling differentiation responses via drug treatment

**Figure 6.**
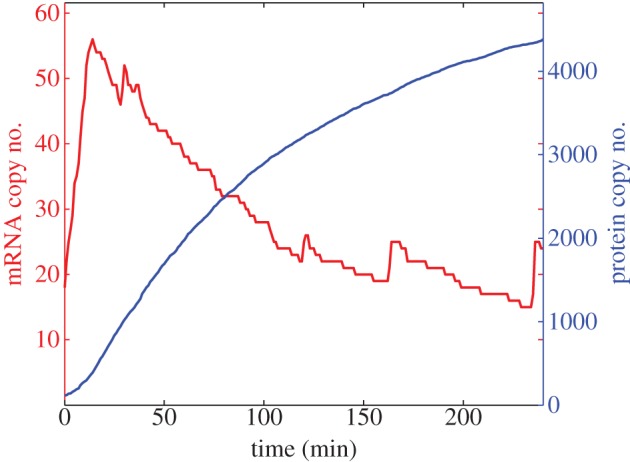
A single trajectory from a proteasome inhibition numerical experiment. The total numbers of hes1 mRNA (red) and Hes1 protein (blue) are plotted against time. Baseline parameter values are used, with the exception of *μ*_p_ which is reduced by a factor 100.

The proteasome is a large proteolytic protein complex found in all eukaryotic cells that is the primary site for degradation of most intracellular proteins. The proteolytic activities of the proteasome can be inhibited by the class of drugs known as proteasome inhibitors. It is known that exposing fibroblast cells to proteasome inhibitors (specifically 100 μM of ALLN) results in increased levels of Hes1 protein and decreased levels of hes1 mRNA. In particular, it was shown that hes1 mRNA levels peak 1 h after proteasome inhibition treatment [1]. We reproduce this experiment using our model by decreasing *μ*_p_ by a factor of 100 and running our simulation for 240 min (figure 6). The model is able to reproduce the experiment qualitatively, i.e. mRNA levels peak quickly then stabilize at a low number while protein levels saturate at high levels. We performed 100 simulations with *μ*_p_ decreased by a factor 100 and found that the average time for hes1 mRNA levels to peak was 29.36 min (shorter than that of fibroblast cells). We are not aware of proteasome inhibition experiments performed in ES cells, and so leave this result as a quantitative prediction of the model. Using our model, we can also make the prediction that ES cells treated with proteasome inhibitors are more likely to differentiate into mesodermal cells.

**Figure 7.**
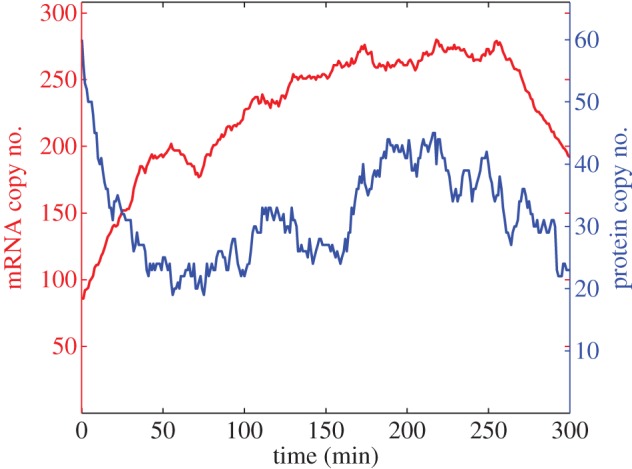
A single trajectory from a translation inhibition numerical experiment. The total numbers of hes1 mRNA (red) and Hes1 protein (blue) are plotted against time. Baseline parameter values are used with the exception of *α*_p_ which is reduced by a factor 100.

Treating cells with cycloheximide inhibits the key process of translation in cells. Experiments have been performed in fibroblast cells to monitor levels of hes1 mRNA in response to this treatment. In the experiments a sustained increase in hes1 mRNA levels is reported [1]. We mimic this experiment with our model by decreasing *α*_p_ by a factor of 100 and running our simulation for 300 min. The results of this numerical experiment are shown in figure 7. Our model is able to reproduce qualitative behaviour, i.e. an increase in hes1 mRNA numbers. In terms of exact numbers, we recorded the mean copy number of hes1 mRNA produced by our model under translation inhibition conditions and compared it with the wild-type case (recording 100 means for each case then taking the average of the means). The translation inhibition experiment caused mean mRNA levels to increase from 50 to 183 (more than threefold increase). We leave this result as a quantitative prediction of the model. Furthermore, we observe that protein levels are persistently low, so using our model we can make the prediction that ES cells undergoing translation inhibition would be more likely to differentiate into neuronal cells.

## 4. Discussion

ES cells are pluripotent stem cells with the ability to differentiate into various cell types belonging to all three germ layers: ectoderm, mesoderm and endoderm. Application of these differentiated cells is highly anticipated for regenerative medicine, but ES cells respond heterogeneously to different cues, resulting in a mixture of various types of differentiated cells. The basic mechanism governing such heterogeneity in the differentiation of ES cells is not well understood but recent studies have suggested the cyclic expression of Hes1 plays a role.

We have presented a spatial stochastic model of the Hes1 GRN that yields results in close agreement with experimental studies. Transcriptional feedback systems in eukaryotic cells are inherently stochastic and spatial and the work presented here emphasizes the need for mathematical models to account for this. With these modelling assumptions, we were able to propose intrinsic noise as the main driving force for the heterogeneity observed in ES cell differentiation responses.

In contrast to recent PDE models of the Hes1 oscillator [28,29], our model is able to reproduce the variability in period and amplitude of Hes1 oscillations observed in experiments. We were able to ask more questions of our model than recent stochastic DDE models [23], as well as being able to directly compare our numerical simulations with bioluminescence movies of *in vivo* Hes1 expression. Additionally, our model does not rely on a Hill function approximation to the negative feedback the Hes1 protein exerts on its own mRNA, the validity of which has been cast into doubt in recent years [43].

Given the potential application for regenerative medicine, we have also proposed methods of controlling differentiation responses via drug treatment. Our model has predicted that applying proteasome inhibitors to an ES cell could yield a mesodermal cell while applying translation inhibitors could yield a neuronal cell. Our model was also able to reproduce experimental results in which hes1 transgenes were introduced to haematopoietic progenitor cell which encoded a mutant Hes1 protein lacking the DNA-binding domain [41].

Future work will consider extending the model in various ways. In particular, we will explicitly account for transport across the nuclear membrane and dimerization of Hes1 monomers. There is experimental evidence that molecular movement within a cell can be ‘subdiffusive’ or ‘superdiffusive’ [44–47], which is something we will investigate in future models. As mentioned earlier, we will also conduct a global sensitivity analysis of our model using data-clustering techniques. We may also consider cell–cell communication in future work to see whether this acts to stabilize and synchronize oscillatory behaviour as Masamizu *et al*. [16] found experimentally and Terry *et al*. [48] found in their model of Notch signalling. Naturally, our approach is readily applicable to many other pathways and future work will investigate the more complex p53–Mdm2 negative feedback loop.
